# Editorial: Pyeloplasties in challenging scenarios as redo procedures, congenital anatomical anomalies and infants: Where do we stand?

**DOI:** 10.3389/fped.2023.1195301

**Published:** 2023-04-17

**Authors:** Simone Sforza, Lorenzo Masieri

**Affiliations:** Department of Pediatric Urology, Meyer Children Hospital, University of Florence, Florence, Italy

**Keywords:** UPJO, pediatric, pyeloplasty, minimally invasive surgery, robotic

**Editorial on the Research Topic** Pyeloplasties in challenging scenarios as redo procedures, congenital anatomical anomalies and infants: Where do we stand?

## Introduction

Uretero-pelvic junction obstruction (UPJO) is the most common congenital ureteral anomaly, occurring in 1 per 20,000 newborns (1). Before the advent of maternal ultrasonography (US), UJPO was usually discovered during evaluation of an abdominal mass, pain or unexplained urinary symptoms in the adult patient (2). In contrast, the incidental detection of UJPO by prenatal US is currently the most common mode of presentation in children (3). This change has resulted in a significant downward shift in the age of pyeloplasty.

Open dismembered pyeloplasty, originally described by Anderson and Hynes in 1949, is the most common surgical procedure performed to treat UJPO either in pediatric and adult's field with a success rate upon 90% (4). In recent years minimally invasive surgery (MIS), as laparoscopy-assisted pyeloplasty (LP) and more recently robot-assisted laparoscopic pyeloplasty (RALP), has emerged as valid alternative to the open surgery also in the younger patients (3, 5, 6).

In particular thanks to the 3 D imagines and the endowrist technologies RALP offers not only the advantages to be minimally invasive but appeared to be the leader technique to perform upper urinary tract reconstructive surgery (7–11). If in the initial phase RALP in pediatric field was confined to school-aged children and adolescent most of the studies describe this subset of population (8, 9, 12, 13), nowadays robotic surgeons have begun to expand its application even in younger infants facing with the challenge of smaller body size and lighter weight (14). Although some authors have already reported encouraging outcomes of RALP in this new group of patients, setting new limits in terms of age and weight, its feasibility is still debated (15, 16).

Moreover, the risk of recurrence exists with a reported percentage of 3%–10% with all of the techniques. Management of UPJO recurrence is more challenging due to scar tissue formation, fibrosis, and decreased vascularization of the ureter tract which necessitate extended resections to find healthy and well-vascularized tissue. In this research topic we explore some important points on UPJO in challenging scenario, as redo pyeloplasty, younger children and associated urological anomalies.

Indeed, Li et al. in this research topic, take a look on the relevant issue of redo pyeloplasty. They review their experience (a total of 453 patients) of redo laparoscopic pyeloplasty (RLP) in patients with recurrent UPJO in comparison to primary LP and redo open pyeloplasty (ROP), and determine the feasibility and effectiveness of RLP for recurrent UPJO in children.

They concluded that RLP performed as well as primary LP except for a longer operation time. Compared with ROP, primary LP has the advantages of a clearer surgical view, sufficient exposure, clearer anatomical landmark position, and minor trauma with a comparable clinical outcome. On experienced hands, primary LP for recurrent UPJO is a safe and effective procedure and should be considered an excellent alternative to the more commonly recommended ROP in select patients.

Similarly, children with congenital anatomical anomalies, such as ectopic kidneys, horseshoe kidneys, or double districts due to the narrower space and fewer landmarks, concomitant urolithiasis are required high experience (17).

Focusing on this aspect Wong et al. published a cases series and a review of the literature on patients with UPJO with concomitant urolithiasis treated with RALP and simultaneous removal of the stones with flexible ureteroscopy; however, only 6 studies worldwide described that type of approach according to the review.

Last but not the least, as well known, high experience is also needed in infants under 1 year or 15 kilograms. Cascini et al. put their attention with a systematic review and meta-analysis on open and MIS pyeloplasty and they evaluate the feasibility and benefits of MIS pyeloplasty compared to OP to surgically treat UPJO in children <1 year of age.

They found nine experiences that meet the inclusion criteria (eight retrospective and one prospective). A total of 3,145 pyeloplasties have been included, with 2,859 (90.9%) OP and 286 (9.1%) MIS. They concluded that MIS presented a longer operative time than OP. However, MIS seemed effective for treating UPJO in infants, showing shortened LOS compared to OP. No differences have been reported with regard to the incidence of postoperative complications and failure of pyeloplasty. Given the low quality of evidence of the meta-analysis according to the GRADE methodology, they suggest limiting MIS procedures in infants to only those high-volume centers with experienced surgeons.

Finally, in our topic, there are reported novel techniques to treat UPJO with long proximal ureteral stricture Han et al. from the group leading by Ce Han or experience dealing with severe hydronephrosis Zhao et al.

They described a surgical modification of the standard Anderson-Hynes techniques on 13 patients mainly including “double-flap” tailoring of the renal pelvis and anastomosis of spatulate ureter with the double-flap. They reported favorable perioperative and postoperative outcomes.

In conclusion, our research topic high light relevant issues in UPJO with a challenging scenario, with two brilliant reviews and 4 well-described monocentric experiences, however, the mare magnum of UPJO in difficult scenario still presents significant aspects to be discovered and evidence to be built.
